# Losing One’s Voice to Save One’s Life: A Brief History of Laryngectomy

**DOI:** 10.7759/cureus.8804

**Published:** 2020-06-24

**Authors:** Boyko Matev, Asen Asenov, George S Stoyanov, Lora T Nikiforova, Nikolay R Sapundzhiev

**Affiliations:** 1 Medicine, Medical University of Varna, Varna, BGR; 2 Department of Otolaryngology, Head and Neck Surgery, Medical University of Plovdiv, Plovdiv, BGR; 3 General and Clinical Pathology/Forensic Medicine and Deontology, Medical University of Varna, Varna, BGR; 4 Otolaryngology, Medical University of Varna, Varna, BGR

**Keywords:** laryngectomy, medical history, conceptual review

## Abstract

Laryngectomy is a surgical procedure that involves the surgical removal of the laryngeal complex, thereby separating the upper from the lower respiratory tracts, resulting in a tracheostomy. In this way, respiration is achieved at the expense of the patient’s voice. A neopharynx is formed, serving only as a digestive passage between the mouth and the esophagus. Until the introduction of the procedure, patients with laryngeal cancer were considered terminally ill. Most often, the title of “First recorded laryngectomy” is held by Theodor Billroth in 1873; however, the outcome of the operation itself was doubtful, with later attempts having a 50% mortality rate. The first major leap in reducing patient mortality rates was the introduction of the two-step laryngectomy, performed by Themistocles Gluck in 1881. This achievement, along with the general advancements in the field of surgery at the time allowed his student Johannes Sørensen to perfect the method and further develop it into a modified single-stage laryngectomy. This procedure is the basis of contemporary methods.

## Introduction and background

A laryngectomy is defined as the surgical removal of all laryngeal structures and a section of the upper trachea. The procedure leads to the formation of a permanent tracheostomy. Indications to perform are the treatment of advanced stages of laryngeal cancer and surgical salvage after failed laryngeal preservation therapies [1]. Laryngectomies as a concept have existed long before the practical means of achieving them. Surgical interventions in the head and neck have been a part of medical practice since the middle ages. The proximity of the vital structures and the laryngeal complex, however, made laryngectomies seem impossible.

Medical science only became advanced enough for surgeons to attempt partial or full laryngectomies in the 19th century. The primary negative consequence of the procedure, however, is aphonia - the inability to produce sounds via the larynx [2]. In conjunction with the loss of speech, protective functions, iatrogenic physical defect, the gravity of the operation, and oncological treatment, the procedure preserves patients’ lives at a significant cost to their quality of life [3].

(This article was presented at the 11th Working meeting of the International Society of History of Otorhinolaryngology 29.09.2017 Varna, Bulgaria.)

## Review

Early attempts and research

The first recorded laryngectomies performed with the specific intention of studying the effects the procedure had on mammals were carried out by Johann Friedrich Hermann Albers (14.11.1805, Dorsten - 11.05.1867, Bonn) in Bonn, Germany in 1829 [4]. He used beagle dogs in his experiments, with one specimen even surviving for 9 days after the procedure. Albers published the first monography on diseases of the larynx, which included over 80 cases of laryngeal illnesses from both personal observations and contemporary sources [5]. There is no record of him performing the procedure on humans, however.

Dogs, while having similar anatomy due to our shared mammalian ancestry, are pronograde, which means that secretions coming from the oropharynx cannot easily enter the trachea, thus rendering them inadequate models [6].

Bernhard Rudolf Konrad von Langenbeck (09.11.1810, Padingbüttel - 29.09.1887, Berlin) was one of the surgeons, responsible for affirming Germany as the world leader in medicine at that time [7-8]. Aside from having a technique named after him (namely the Von Langenbeck cleft palate repair technique), he was also responsible for the medical education of historically significant surgeons such as Theodor Billroth and Themistocles Gluck among his many other achievements [7]. Some sources affirm that Von Langenbeck had indeed proposed such an operation in 1854 [9].

First alleged laryngectomy

A great deal of misconception has existed for years concerning the contribution of Sir Patrick Heron Watson (05.01.1832, Edinburgh-21.12.1907, Edinburgh) towards the history of laryngectomy [10]. Sir Watson's brilliant career has left its mark even beyond the borders of Britain. He served as a doctor during the Crimean War, in the same hospital as Florence Nightingale, who was herself recovering from an attack of Crimean fever and even wrote to him and his brother while they had to be hospitalized due to dysentery there:

“Miss Nightingale is very sorry she cannot come to see you” [11].

At home, he was a key part of the introduction of anesthetics.

The controversy surrounding Watson originates from a case of his in 1866 of a person with a larynx, severely deformed and damaged by tertiary syphilis [12]. Watson himself never publicly presented the case; however, he did disclose it in personal correspondence with David Foulis, who, in 1881 decided to publish it [13-14]. Foulis rather ambiguously describes the case as extirpation of the larynx and awards the procedure of Watson with the title of the first laryngectomy (usually reserved for Theodor Billroth’s operation, some 11 years after the aforementioned event). Foulis' methods, published alongside Watson's procedure, were severely criticized by his peers [15-18].

There are, however, reasons to dispute Foulis' claim. First, in a general sense, a laryngectomy is a procedure, reserved for the treatment of laryngeal cancer, rather than the advanced stages of syphilis. However, it is feasible to imagine that untreated syphilis in the 19th century would damage the tissues of the larynx enough to necessitate removal. A more solid argument against the statement is presented by the records of Watson himself, who never explicitly states that he had removed the larynx, merely pieces of it. A laryngectomy in the literal sense was performed post-mortem, to examine the unusually damaged organ [19].

The first recorded laryngectomy

Christian Albert Theodor Billroth (26.04.1829, Bergen auf Rügen - 06.02.1894, Opatija) is perhaps one of the most venerated surgeons in modern medicine [20]. Widely recognized by his peers for his skills and resourcefulness, he was eventually dubbed “surgeon of great initiatives”, even after a lackluster early education and an incident of a rather spectacularly failed gastric surgery in Vienna that nearly led to him being stoned to death [21].

Billroth I, the removal of the lower portion of the stomach (pylorus) with end-to-end anastomosis of the remaining stomach with the duodenum, and Billroth II, gastrojejunal anastomosis with duodenal closure, are named after him [20].

One of the other reasons why he is so highly esteemed even to this day is the laryngectomy he performed, which is considered as the first one. The surgery took place on the 31st of December 1873 on a 36-year-old religious tutor with a tumor below the true vocal cords. While the operation started only as partial removal of the larynx, further examination proved the necessity of removing the entirety of the organ. Billroth had even planned for the construction of an artificial larynx. After the successful operation and turbulent recovery period, the patient survived for seven months, before passing away due to a local recurrence [22].

While the operation was a resounding success, and other medical centers incorporated it into their practice, surgeons soon realized the disadvantages of the procedure. Statistics almost uniformly yielded a 50% mortality rate, leading practitioners to recommend the surgery exclusively as a last resort [23]. Billroth himself stated:

“What do people really know of my scientific accomplishments? Nothing. A myth develops into a miracle through the imagination of the people. I believe the surgical removal of the larynx and replacing it with an artificial one was the beginning of the myth about me” [24].

The first two-stage laryngectomy

Contrary to Billroth's night legendary status as a surgeon, Themistocles Gluck (30.11.1853, Iaşi - 25.04.1942, Berlin) was heavily neglected by his contemporary sources, receiving much more recognition from non-German peers and non-surgeons from Germany [25]. This was mostly due to Ernst von Bergmann's (16.12.1836, Riga - 25.03.1907, Wiesbaden), the successor to von Langenbeck and a major authority among German surgeons’, contempt for his progressiveness, who at one point stated:

“…my pupils and I will fight you with all means” [26].

The main cause of postoperative death after a laryngectomy was aspiration pneumonia. Gluck devised a two-stage operation to reduce aspiration: the trachea was severed from the larynx and sutured to the skin, establishing a tracheostomy. Two weeks later the larynx was resected, and the pharyngeal defect was closed. The procedure was published in 1881. While the operation was still far from refined, being difficult and time-consuming, the innovations, introduced by Gluck reduced the mortality rates at least tenfold [27-28].

While he made modern laryngectomies possible, Gluck, even to this day receives very little recognition for this feat. Instead, he is more commonly associated with advances in endoprosthesis, even having the annual award for developments in endoprosthesis by the German Society for Orthopaedic Surgery named after him in 2000 [27].

Transition to a single-stage laryngectomy

If Gluck is all but ignored by medical literature of his time, the person regarded as his assistant - Johannes Sørensen (1862-1939) is indeed entirely forgotten. While he was a subordinate to Gluck, he was also a close confidant and friend, who independently improved the technique.

Gluck and Sørensen together developed a single-stage operation, introducing tracheal dissection at the end of the procedure, reducing the risk of local infection with a permanent pharyngeal closure, accomplished in parallel.

This improvement to the technique in 1890 led to a significant drop in mortality rates (as low as 4%). By 1922, Gluck and Sørensen had completed 160 such procedures, the last consecutive 63 of which were without a lethal outcome, thus creating the basis for most contemporary laryngectomies [27].

This was of course not the final chapter of the history of this procedure. Its evolution in the last two centuries and the introduction of antibiotics led to many improvements and in the technique before being standardized and widely accepted as the gold standard today [29]. Contemporary guidelines in cancer treatment renounce the operation as a standalone procedure in advanced cancer and often prescribe the execution of chemotherapy and radiotherapy in unison with it. This is in alignment with the general philosophy that led to the birth of the operation in the first place - a sacrifice in the quality of life of the patient, to increase survivability [30].

Contemporary methods

The “da Vinci Surgical System” (Intuitive Surgical, Inc., Sunnyvale, CA) has routinely been used in abdominal and urological surgeries since 2000. The first transoral robot-assisted surgery (TORS) trials were conducted in 2006. While the procedure is still somewhat new, and its implementation is made difficult by the relatively expensive equipment and the necessary additional training for the operators, the method will likely see increased use in the future [31]. Narrow-field total laryngectomy by way of TORS has been reported to be successful in patients, however, the application of partial laryngectomies is still a matter of discussion [32]. Canine model testing with commercially available systems (namely the da Vinci Surgical Robot) showed promise and a comparison of treatment outcomes of TORS and supraglottic partial laryngectomy rule in favor of the former as far as patient recovery time and quality of life [33-34].

## Conclusions

The laryngectomy has indeed come a long way since the early days of its introduction. It has a rich and somewhat controversial history. Several procedures can be dubbed as "the first laryngectomy" and it is up to the discretion of the reader to decide whichever fits their subjective understanding of "first" best.
